# Long-term exposure to PM_2.5_ leads to mitochondrial damage and differential expression of associated circRNA in rat hepatocytes

**DOI:** 10.1038/s41598-024-62748-y

**Published:** 2024-05-24

**Authors:** Ying Liu, Jing Li, Yican Xiong, Chaochao Tan, Cunyan Li, Youde Cao, Wanying Xie, Zhonghua Deng

**Affiliations:** 1 Department of Medical Laboratory, Hunan Provincial People’s Hospital, the First Affiliated Hospital of Hunan Normal University, Changsha, 410005 People’s Republic of China; 2 Department of Ophthalmology and Stomatology, Hunan Provincial People’s Hospital, the First Affiliated Hospital of Hunan Normal University, Changsha, 410005 People’s Republic of China

**Keywords:** Environmental sciences, Non-coding RNAs

## Abstract

Fine particulate matter (PM_2.5_) is one of the four major causes of mortality globally. The objective of this study was to investigate the mechanism underlying liver injury following exposure to PM_2.5_ and the involvement of circRNA in its regulation. A PM_2.5_ respiratory tract exposure model was established in SPF SD male rats with a dose of 20 mg/kg, and liver tissue of rats in control group and PM_2.5_-exposed groups rats were detected. The results of ICP-MS showed that Mn, Cu and Ni were enriched in the liver. HE staining showed significant pathological changes in liver tissues of PM_2.5_-exposed group, transmission electron microscopy showed significant changes in mitochondrial structure of liver cells, and further mitochondrial function detection showed that the PM_2.5_ exposure resulted in an increase in cell reactive oxygen species content and a decrease in mitochondrial transmembrane potential, while the expression of SOD1 and HO-1 antioxidant oxidase genes was upregulated. Through high-throughput sequencing of circRNAs, we observed a significant down-regulation of 10 and an up-regulation of 17 circRNAs in the PM_2.5_-exposed groups. The functional enrichment and pathway analyses indicated that the differentially expressed circRNAs by PM_2.5_ exposure were primarily associated with processes related to protein ubiquitination, zinc ion binding, peroxisome function, and mitochondrial regulation. These findings suggest that the mechanism underlying liver injury induced by PM_2.5_-exposure may be associated with mitochondrial impairment resulting from the presence of heavy metal constituents. Therefore, this study provides a novel theoretical foundation for investigating the molecular mechanisms underlying liver injury induced by PM_2.5_ exposure.

## Introduction

With the acceleration of the process of modernization industrialization, air pollution is increasing and is seriously threatening human health. Among various air pollutants, particulate matter (PM_2.5_) has the greatest impact on humans. PM_2.5_ refers to particulate matter with an aerodynamic diameter of ≤ 2.5 μm in environmental pollution particles^1^. The 2019 Global Burden of Disease study shows that 86.5 deaths per 100,000 people are caused by exposure to PM_2.5_ and ranks it as the fourth leading risk factor for death and disability-adjusted life years globally^2^. Due to its large surface area, PM_2.5_ can adsorb various harmful substances into human body, such as polycyclic aromatic hydrocarbons and heavy metals. These substances enter various organs through the bloodstream, and heavy metals, being difficult to degrade, deposit in organs such as the liver and kidneys after entering the body, causing organ damage^3,4^. Therefore, prolonged or severe PM_2.5_ exposure leads to liver injury.

A meta-analysis has shown that sustained PM_2.5_ exposure significantly increases the risk of chronic liver disease^5^. Epidemiological evidence indicates that for every 10 μg/m^3^ increase in ambient PM_2.5_ exposure, the risk of liver cancer increases by 26%^6^. Currently, biomarkers such as liver enzymes play an important role in indicating the severity of liver injury as indicators of liver function. Our previous big data analysis indicate a stable correlation between PM_2.5_ and various liver biomarkers, such as total protein (TP), alanine aminotransferase (ALT), aspartate aminotransferase (AST), total bilirubin (TBIL), and direct bilirubin (DBIL)^7^. Research evidence suggests that exposure to PM_2.5_ leads to elevated levels of liver enzymes, especially ALT, as a marker of liver injury. The changes in ALT levels can indicate the severity of liver injury, and it also serves as an independent predictor for hepatocellular carcinoma (HCC)^8,9^. In a cohort study by Deng et al., 17,106 subjects were used to reveal the relationship between the exposure to PM_2.5_ and nonalcoholic fatty liver disease (NAFLD). The results showed that the risk of NAFLD in adults is directly proportional to long-term PM_2.5_ exposure^10^. Similarly, exposure to PM_2.5_ increases the levels of reactive oxygen species (ROS) in vivo, as observed in experimental results by Ding and Xu et al., confirming that PM_2.5_ exposure can aggravate oxidative stress and inflammatory responses in hepatocytes, resulting in liver injury^11,12^. However, to date, the molecular regulatory mechanism of liver injury caused by PM_2.5_ remains unclear. Studies have reported that after the heavy metal components carried by PM_2.5_ enter the human body and circulate to the liver, they can induce oxidative stress by inhibiting the activity of various biological enzymes in the liver, causing liver cell damage and ultimately liver dysfunction^13^. Therefore, oxidative stress may be the main mechanism of liver damage caused by PM_2.5_ exposure.

Oxidative stress is caused by the imbalance between the generation and clearance of ROS, which attacks human cells, tissues and other structures, resulting in functional damage of corresponding cells, tissues and other structures. Mitochondria are not only the sites of energy production and metabolism, but also the main sources and targets of ROS ^14^, playing an important role in maintaining the balance between oxidation and antioxidant. Mitochondrial structural damage and dysfunction can damage the integrity of tissues, and is closely related to many human diseases. ROS can be generated through a mechanism similar to positive feedback. On the one hand, mitochondria can produce ROS; On the other hand, the ROS produced can in turn damage the mitochondria, causing the damaged mitochondria to produce more ROS. However, ROS can trigger mitochondrial autophagy and selectively remove structurally and functionally damaged mitochondria, thereby reducing ROS levels in vivo^15–17^. Mitochondrial autophagy is a mechanism that protects cells from ROS damage in vivo and plays an important role in maintaining ROS balance in vivo. Evans et al. induced mitochondrial autophagy to remove damaged mitochondria through autophagosome isolation and phagocytosis, thereby reducing the production of ROS^18^. Studies have confirmed the relationship between PM_2.5_ exposure and liver mitochondrial damage: PM_2.5_ exposure induces liver mitochondrial stress and enhances inflammatory response by increasing oxidative stress ROS levels in the body, ultimately leading to liver damage^19,20^. Studies have shown that circRNA can mediate the production of ROS to regulate oxidative stress, and participate in the process of liver injury by regulating mitochondrial autophagy^21,22^. Therefore, we speculate that circRNA may participate in the liver injury process caused by PM_2.5_ exposure through regulating oxidative stress.

Circular RNAs (circRNAs) are a class of non-coding RNAs (ncRNAs) with closed-loop structures that act sponges for competitive endogenous RNAs (ceRNAs) and microRNAs (miRNAs), regulate the expression of downstream genes, and are stably and widely expressed in different species^23,24^. The results of studies showed that circRNAs can be used as diagnostic markers for various diseases^25^. Recently, several researchers demonstrated that PM_2.5_ exposure leads to differential circRNA expression in lung tissues and cells^26,27^. In addition, 25 circRNAs were downregulated and 55 circRNAs were upregulated in rat embryos exposed to high concentrations of PM_2.5_ and differentially expressed circRNAs (DEcircRNAs) may be associated with air pollution-related congenital malformations based on Gene Ontology (GO) and Kyoto Encyclopedia of Genes and Genomes (KEGG) analysis^28^. In addition, circRNA was found to inhibit mitochondrial ROS production and alleviate cirrhosis^29^. Although circRNA has been confirmed to be differentially expressed in a variety of organs after PM_2.5_ exposure, especially the lung, the studies on differentially expressed in the liver after PM_2.5_ exposure have been relatively rarely reported at home and abroad.

In this study, we established an experimental model of PM_2.5_ exposure of rats’ respiratory tracts. Liver samples were collected and subjected to hematoxylin–eosin staining (HE) for histopathological observation, while transmission electron microscopy was employed to observe the ultrastructural changes in the liver cells. High-throughput sequencing of circRNA combined with bioinformatics analysis technology was utilized to identify and analyze the potential biological functions associated with DEcircRNA.

## Materials and methods

### PM_2.5_ Preparation

PM_2.5_ (SRM 1648a) used in this study was purchased as a standard reference material certified by the National Institute of Standards and Technology (NIST). PM_2.5_ required for the experiment was weighed and dissolved in 0.9% normal saline (NS) to prepare a 6.67 mg/mL stock solution and stored at 4 °C. Before use, the suspension was sonicated for 30 min to disperse the particles before autoclaving^13^. The content of 9 metals (As, V, Cr, Mn, Cu, Ni, Pb, Hg, Co) in PM_2.5_ was analyzed by inductively coupled plasma-mass spectrometry (ICP-MS, Thermo Fisher Scientific, Massachusetts, USA) and the physical properties of PM_2.5_ were measured using the Litesizer 500 (Anton Paar, Austria) analyzer.

### Animals

Twenty male specific pathogen free (SPF) Sprague–Dawley (SD) rats, aged 6–8 weeks and weighing 180–200 g were purchased from Hunan SJA Laboratory Animal Co., Ltd. [SCXK (Xiang) 2019–0004] and raised in the animal barrier environment of Hunan Provincial People’s Hospital [SYXK (Xiang) 2013–0003], with normal diet and drinking water, alternating 12 h light and dark, and an ambient temperature varying from 22–24 °C. The study protocol was reviewed and approved by the Ethics Committee of Hunan Provincial People ^'^s Hospital. All experiments are conducted in accordance with the guidelines and regulations of the ARRIVE Guide.

### Animal exposure

After one week of adaptive feeding, 20 rats were randomly divided into control and PM_2.5_-exposed experimental groups. Each group contained 10 rats. After isoflurane anesthesia, an experimental animal model of PM_2.5_ exposure was constructed by tracheal instillation. The PM_2.5_ exposure dose was 20 mg/kg in the experimental group and 0.9% NS was used in the control group. The instillation dose of 3 mL/kg body weight in the two groups^30^ was administered once a week for 16 weeks. Rats were sacrificed one week after the last exposure and liver tissues were frozen in liquid nitrogen until use. The concentration limit of PM_2.5_, an environmental pollutant in China, is no more than 75 μg/m^3^ on average for 24 h. Adult rats weighed 250 g and had a respiratory volume of 0.105 m^3^/day. Therefore, the PM_2.5_ inhalation amount of each rat was 7.875 μg/day (0.105 m^3^/day × 75 μg/m^3^). Considering the uncertainty of interspecific differences (10–100-fold)^31^, the dose used in this study was set as 20 mg/kg (7.875 μg/day × 7 day × 100-fold /250 g)^32^.

### Liver tissue digestion and metal content detection

Six rats (3 in the control group and 3 in the PM_2.5_ exposure group) were randomly selected to take 0.1 g of liver tissue (accurate to 0.001 g), 1 mL concentrated nitric acid and 3 mL hydrochloric acid for microwave digestion. After cooling, the digestion solution was transferred to a 50 mL volumetric bottle, and the volume was diluted with ultra-pure water to the scale for metal content detection. The contents of lead, arsenic, manganese, copper, mercury, cadmium, cobalt, vanadium and nickel in liver tissue were analyzed by ICP-MS. The test parameters of ICP-MS are shown in Table 1.

**Table 1:** ICP-MS test parameter settings.

Argument	Settings
Atomizing gas flow (L/min)	0.70
Plasma gas flow (L/min)	12.0
Auxiliary gas flow (L/min)	1.00
Radio frequency power (kW)	1.20
Observed altitude (mm)	8
Observation mode	Radial direction
Settling time (s)	15
Number of repeats	3

### Identification of liver histopathological morphology

The liver tissues of six rats (3 in control group and 3 in PM_2.5_ exposure group) were randomly selected and fixed in 4% paraformaldehyde overnight. In order from low concentration to high concentration, the fixed tissues were dehydrated in ethanol. The dehydrated tissue blocks were placed in xylene to remove ethanol. It is then embedded in paraffin wax. A microtome was used to cut the paraffin-embedded liver tissue into 6 μm slices for hematoxylin–eosin staining, observation and image collection under an optical microscope^33^.

### Ultrastructural observation of hepatocytes by *electron* microscope

The liver tissues of six rats (3 in the control group and 3 in the PM_2.5_ exposure group) were randomly selected and fixed with 2.5% glutaraldehyde for 6 h, followed by 1% osmium tetroxide for 1 h. The tissue fixation blocks were dehydrated in acetone and then embedded in epoxy resin. The embedded tissue blocks were cut into 70 nm slices using an ultra-thin microtome (Leica EMUC7, Germany). After staining with 4% uranyl acetate and lead citrate, mitochondrial morphology was observed on copper grid by transmission electron microscopy (HT7800, Hitachi, Japan)^34^. Mitochondrial number, diameter, and area were measured using Image J software.

### Cell culture

BRL 3A cells (Wuhan Punocai Life Technology Co., LTD.) were inoculated in ATCC modified MEM complete medium containing 10% fetal bovine serum and 1% penicillin streptomycin double antibody, placed in a 5% CO2 cell incubator at 37 ℃, and passed when the cells were fused to 70–80%.

### Cell ROS detection

Inoculated the six-well plate with 2.5 × 10^5^/mL cells per well. After cell growth and fusion to 70–80%, 100 ug/mL^35^ PM_2.5_ venom containing complete culture medium was placed in a cell incubator at 37 ℃ for 24 h. The PM_2.5_ venom was absorbed, H2DCFDA working solution was added, and incubated in a cell incubator at 37 ℃ in dark light. After 30 min, the serum free culture solution was washed 1–2 times. After completion, the fluorescence microscope was used for observation, and the fluorescence intensity was analyzed by Image J software.

### Mitochondrial transmembrane potential detection

Cell inoculation and treatment are the same as the above methods. Culture solution was removed, cells were washed with 1xPBS solution, 1 mL cell culture solution and JC-1 working solution were added, and incubated at 37 ℃ for 20 min. After incubation, the cells were washed with buffer solution. The cell culture medium was added and observed under fluorescence microscope, and the fluorescence intensity was analyzed by Image J software.

### Total RNA extraction

Total RNA was extracted from six randomly selected samples (three control and three PM_2.5_-exposed experimental groups) using TRIzol reagent (Invitrogen, Thermo Fisher Scientific, USA). The total RNA purity was measured using a NanoDrop 2000 spectrophotometer (Thermo Fisher Scientific, USA) and optical density (OD) 260/280 between 1.8 and 2.1 was considered to be acceptable. The total RNA integrity was verified by Agilent 2100/2200 Bioanalyzer (Agilent Technologies, USA) and agarose gel electrophoresis.

### RNA sequencing

rRNA subtractive libraries (NEBNext® Ultra™ Directed RNA Library Preparation Kit) were constructed according to the manufacturer’s instructions. The entire transcriptome sequence sequenced by the HiSeq™ Sequencer was screened (adapter sequences were removed, ambiguous bases with reads > 5% (denoted as N), low-quality reads containing more than 20% bases with a quality < 20) and mapped to the human genome using HISAT 2 (GRCh38 NCBI). High-throughput sequencing (HT-Seq) was used to calculate the number of genes.

### Differential gene expression analysis

HT-seq data were analyzed using the DEseq algorithm to screen for circRNAs that were differentially expressed in liver tissue from the control versus PM_2.5_-exposed experimental rats, with a threshold of | log_2_FoldChange | > 0.585, *P* < 0.05.

### DEcircRNA target gene prediction and circRNA function analysis

The miRanda package was introduced into full-length sequences of DEcircRNAs to predict highly matched miRNA-binding sites. GO (http://geneontology.org/) and KEGG (https://www.genome.jp/kegg/) were used to perform gene ontology analysis, pathway analysis, and functional annotation of DEcircRNAs.

### RT-qPCR

Total RNA was extracted from rat hepatocytes using TRIzol reagent (TKR-9108, Takara, Japan) according to manufacturer's instructions. A reverse transcription reaction was then performed using a reverse transcription reagent with gDNA Eraser (TKR-RR047A, Takara, Japan) to reverse transcribe total RNA into cDNA, and finally using TB Green® Premix Ex Taq™ reagent (RR420A, Takara, Japan). Japan) RT-qPCR was performed on Step One Plus (Applied Biosystems, Carlsbad, CA, USA). *β*-actin is used as an internal reference gene to correct relative gene expression. The relative expression was calculated by 2^-ΔΔCt^. The primer sequences used in the study are supplemented in Supplementary Table S1.

### Statistical analysis

SPSS 25.0 software is used for statistical analysis. Count data conforming to the normal distribution were presented as mean ± standard deviation and the difference between the two groups was compared using Student’s *t*-test. If the normal distribution does not conform, the Mann–Whitney non-parametric test is used. Significant *P*-values were defined by Fisher’s exact test and *P*-values less than 0.05 were considered to be statistically significant.

### Ethics approval and consent to participate

The experimental protocol has been approved by the Ethics Committee of Hunan Provincial People's Hospital. All individuals volunteered to participate in the study and provided informed written consent for participation. I promise that the study was performed according to the international, national and institutional rules considering animal experiments, clinical studies and biodiversity rights.

## Results

### Characteristics of PM_2.5_

We analyzed the content of PM_2.5_ used in the experiment, which mainly included nine metals (As, V, Cr, Mn, Cu, Ni, Pb, Hg, Co), and the metal content of water and feed were also analyzed, as shown in Supplementary Table S2. At the same time, the physical characteristics of PM_2.5_ were tested, and the results showed that the average particle size of PM_2.5_ was 912.2 ± 447.8 nm (Supplementary Fig. S1), and the polymer dispersion index (PDI) was 29.56%, indicating that PM_2.5_ was uniformly dispersed in the system (the smaller the PDI value, the more uniform the molecular weight distribution of the polymer; otherwise, the wider the distribution). The above results show that the particle size and distribution of PM_2.5_ conform to the experimental standards.

### Metal content in liver tissue

**Figure 1:**
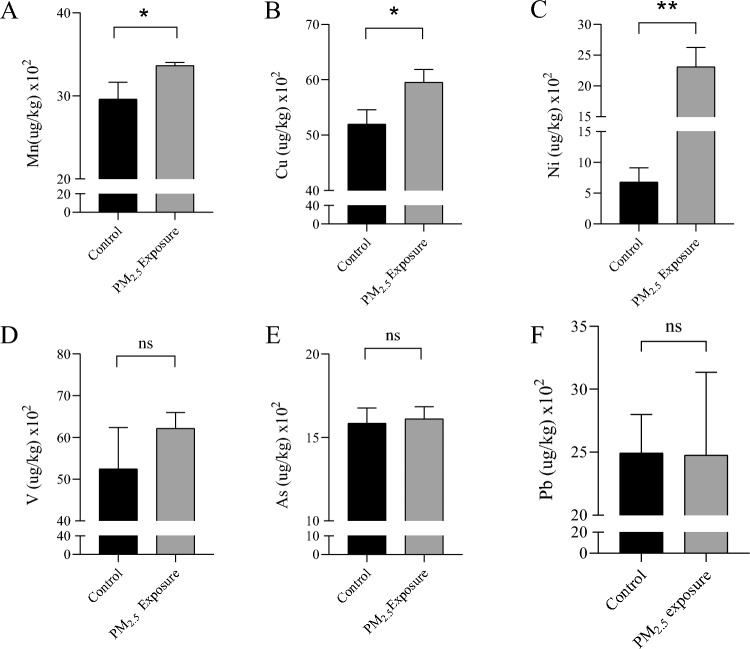
Content of metal in liver tissue. (**A**) Content of Mn in liver tissue. (**B**) Cu content in liver tissue. (**C**) Ni content in liver tissue. (**D**) The amount of V in liver tissue. (**E**) Content of As in liver tissues. (**F**) Content of Pb in liver tissues (data represent mean ± standard deviation, *: *P* < 0.05, **: *P* < 0.01, ns: *P* > 0.05).

In order to detect the content of metal components carried by PM_2.5_ in the liver, we used ICP-MS to detect the content of nine metals: lead, arsenic, manganese, copper, mercury, cadmium, cobalt, vanadium and nickel. Results As shown in Fig. 1A–C, among the nine metals, the content of Mn, Cu and Ni in PM_2.5_ exposure group was significantly higher than that in control group (*P* < 0.05). There was no statistical significance in the contents of V, As and Pb between the two groups (Fig. 1D–F). Hg, Co and Cd were below the detection limit and were not detected.

### Histopathological changes of liver

**Figure 2:**
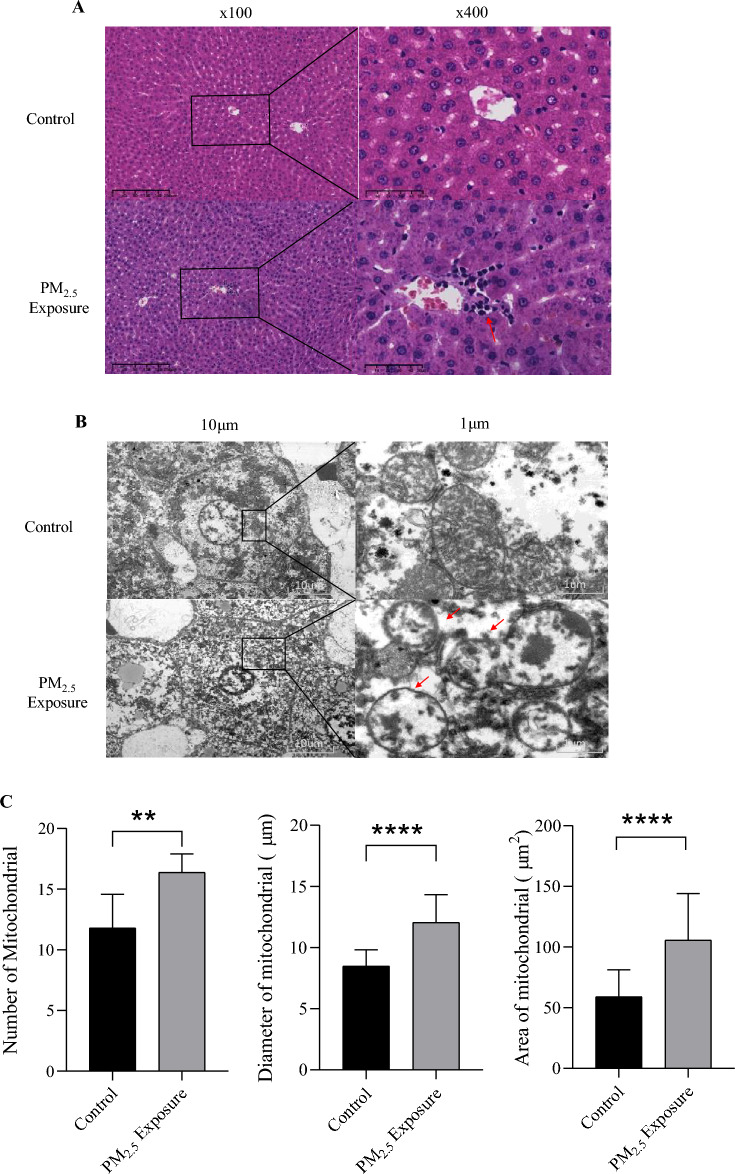
Pathological changes of liver tissue in rats induced by PM_2.5_ exposure. (**A**) Optical microscope was used to observe the liver tissue sections stained by HE. The control group and the PM_2.5_ exposure group were imaged at 100 × and 400 × magnification, respectively. The red arrow showed inflammatory cell infiltration. (**B**) Transmission electron microscopy (TEM) of control group and PM_2.5_ exposure group showed mitochondrial structure of liver (magnification of 10 μm and 1 μm). (** C**) Number, diameter and area of mitochondria. **: *P* < 0.01, ****: *P* < 0.0001.

To verify whether PM_2.5_ exposure caused histopathological changes in liver tissue, hematoxylin–eosin staining was performed on animal liver tissue. As shown in Fig. 2A, compared with the control group, the liver morphology of rats in the PM_2.5_ exposure group was significantly changed, which was mainly manifested as the destruction of the liver lobular structure and the disordered cell arrangement, especially the clumped and dark-colored inflammatory cells. Figure 2B shows the mitochondrial structure of liver tissue. Compared with the control group, the morphology of liver mitochondria in the PM_2.5_ exposed group showed significant changes, including blurred internal structure of mitochondria, fusion of outer membrane and inner membrane, disappearance of membrane gap, mitochondrial ridge dissolution, and even fusion of two mitochondria into one. The number, diameter, and area of mitochondria increased in the PM_2.5_ exposed group compared with the control group (Fig. 2C). By observing the results of HE staining and transmission electron microscopy of the control group and the PM_2.5_ exposure group, we found that the morphology and ultrastructure of the liver tissues in the PM_2.5_ exposure group were significantly changed, which preliminarily indicated that PM_2.5_ exposure caused damage to liver tissues and liver mitochondria. More image information is visible in the Supplementary Fig. S2.

### Detection of mitochondrial function and oxidative stress gene expression

**Figure 3:**
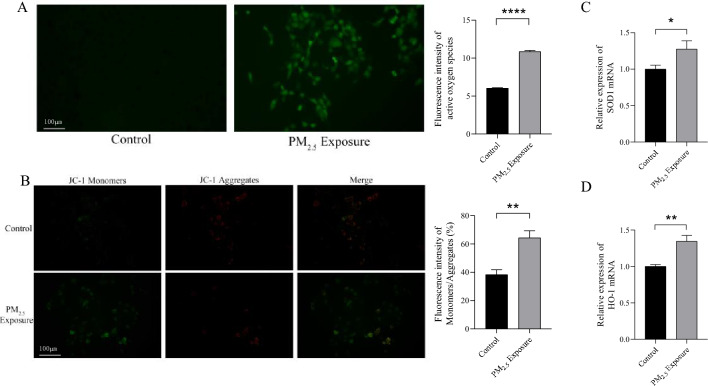
Mitochondrial function and oxidative stress gene detection in rat cells. (**A**) The fluorescence intensity of ROS in liver cells of rats in control group and PM_2.5_ exposure group (100x). Green fluorescence indicates the detection of intracellular reactive oxygen species. (**B**) Mitochondrial membrane potential results of control group and PM_2.5_ exposure group (100x). Red fluorescence is emitted when the mitochondrial membrane potential is normal, and green fluorescence is emitted when the mitochondrial membrane potential is decreased. (**C**) SOD1 mRNA levels in control group and PM_2.5_ exposure group. (**D**) HO-1 mRNA levels in control group and PM_2.5_ exposure group. *: *P* < 0.05, **: *P* < 0.01, ****: *P* < 0.0001.

In order to verify whether PM_2.5_ exposure causes intracellular oxidative stress and mitochondrial dysfunction, we examined markers and genes involved in these processes. Cellular ROS tests showed that the levels of ROS in the PM_2.5_ exposure group were significantly higher than those in the control group (Fig. 3A). Compared with the control group, the mitochondrial transmembrane potential of PM_2.5_ exposure group was significantly decreased (Fig. 3B), the fluorescence intensity of mitochondrial transmembrane potential monomers (green) was significantly enhanced, the fluorescence intensity of mitochondrial transmembrane potential aggregates (red) was significantly decreased, and the percentage of fluorescence intensity of monomers and aggregates was significantly increased. In addition, RT-qPCR was also performed on oxidative stress-related genes, and the results showed that the relative expression levels of SOD1 mRNA and HO-1 mRNA in the PM_2.5_ exposure group were higher than those in the control group (Fig. 3C and D) (*P* < 0.05 or *P* < 0.01). These results suggest that PM_2.5_ exposure causes damage to mitochondrial function in hepatocytes.

### Identification of circRNA in rat liver tissue

**Figure 4:**
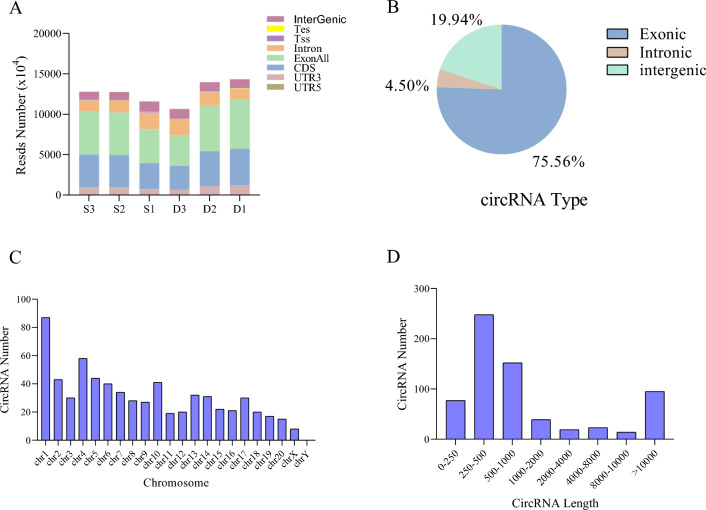
circRNA identification of rat liver. (**A**) Number of different gene structures in 6 liver tissues (**B**) circRNA type distribution in rat liver tissues. (**C**) circRNA distribution on chromosomes. (**D**) Length of circRNA.

To investigate whether air-polluted particulate matter PM_2.5_ expresses circRNA in rat liver tissue, we performed whole-transcriptome sequencing on six randomly selected tissue samples. The original sequence data can be accessed through https://ngdc.cncb.ac.cn/gsa/s/D5uC3wSR. The results of agarose gel electrophoresis and Agilent 2100/2200 bioanalyzer showed good integrity of RNA. (Supplementary Fig. S3, S4). According to the results of sequencing, the structure of all genes in the six tissues is mainly exons, coding sequences (CDS) and introns (Fig. 4A). A total of 667 circRNAs expressions were identified in the control and experimental groups, with exon circRNAs accounting for the majority, which were higher than intronic circRNAs and intergenic circRNAs (Fig. 4B). As shown in Fig. 4C, circRNAs were distributed on almost all chromosomes, with chromosome 1 being the most abundant and almost no distribution on chromosome Y. In the control and experimental groups, most circRNAs were concentrated in the 250-500 bp in length (Fig. 4D).

### DEcircRNAs in rat liver during PM_2.5_ exposure

**Figure 5:**
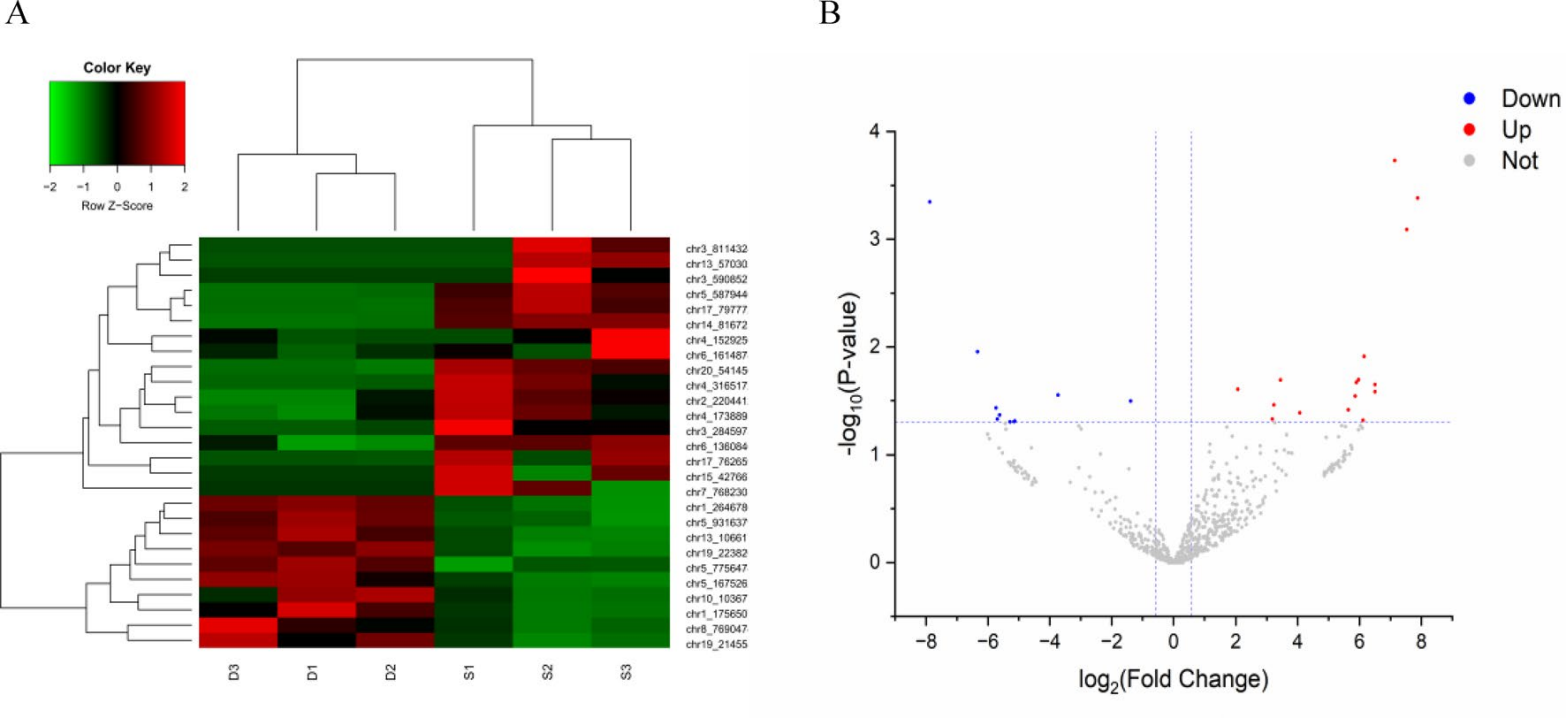
Differentially expressed circRNAs in rat liver tissue. (**A**) Cluster heatmap of differentially expressed circRNAs. Green and red represent downregulated and upregulated circRNAs, respectively. (**B**) Volcano plot of circRNA differential expression. Blue and red indicate 1.5-fold down- and upregulated circRNAs, respectively.

DEcircRNAs in the rat liver were screened using the DEseq algorithm. By comparing the sequencing data between the control and PM_2.5_ exposed groups, we identified 27 circRNAs differentially expressed in the PM_2.5_-exposed experimental group according to the screening criteria (| log_2_FoldChange | > 0.585, *P* < 0.05), including 10 downregulated circRNAs and 17 upregulated circRNAs (Table 2). To determine the potential relationship between the two groups, clustered heat maps (Fig. 5A) and volcano maps (Fig. 5B) were used to illustrate DEcircRNAs.

**Table 2:** Differentially expressed circRNAs in rat liver tissue.

circID	Host Gene	Style	log_2_FC	*P*值
chr19:22382073–22373973	Esrrg	Down	− 5.29	0.0499
chr1:175650721–175646900	Lonp2	Down	− 5.62	0.0431
chr5:167526275–167516581	Itfg1	Down	− 5.15	0.0497
chr5:93163795–93160495	rnf141	Down	− 5.73	0.0370
chr8:76904745–76897417	Rere	Down	− 6.33	0.0111
chr10:103677527–103586432	Ptprd	Down	− 3.73	0.0281
chr5:77564788–77247680	Rnf111	Down	− 5.70	0.0469
chr1:264678002–264674065	Cd300lf	Down	− 7.88	0.0004
chr19:22382073–22373973	Mup4	Down	− 1.38	0.0320
chr1:175650721–175646900	Slf2	Down	− 5.13	0.0489
chr4:173889303–173885673	Pik3c2g	Up	2.07	0.0248
chr2:220441299–220435620	Frrs1	Up	3.24	0.0346
chr6:1614878–1613963	Prkd3	Up	3.18	0.0469
chr4:152925673–152921913	Kdm5a	Up	4.07	0.0410
chr6:136084622–136059672	Mark3	Up	3.45	0.0203
chr14:81672500–81667646	Rnf4	Up	5.90	0.0214
chr15:42766794–42766683	Ephx2	Up	7.53	0.0008
chr17:76265943–76250434	Upf2	Up	5.64	0.0385
chr17:79777211–79740146	Mindy3	Up	7.14	0.0002
chr3:28459755–28452153	Kynu	Up	5.86	0.0287
chr4:31651720–31608269	Slc25a13	Up	5.96	0.0202
chr5:58794404–58792581	Atp8b5p	Up	6.15	0.0123
chr7:76823032–76818801	Ubr5	Up	6.12	0.0481
chr20:5414567–5375281	RT1-A2	Up	11.54	0.0000
chr13:57030242–57029508	Cfh	Up	7.88	0.0004
chr3:81143240–81127393	Phf21a	Up	6.50	0.0224
chr3:59085278–59075644	na	Up	6.50	0.0261

### Functional enrichment analysis of differentially expressed circRNAs

**Figure 6:**
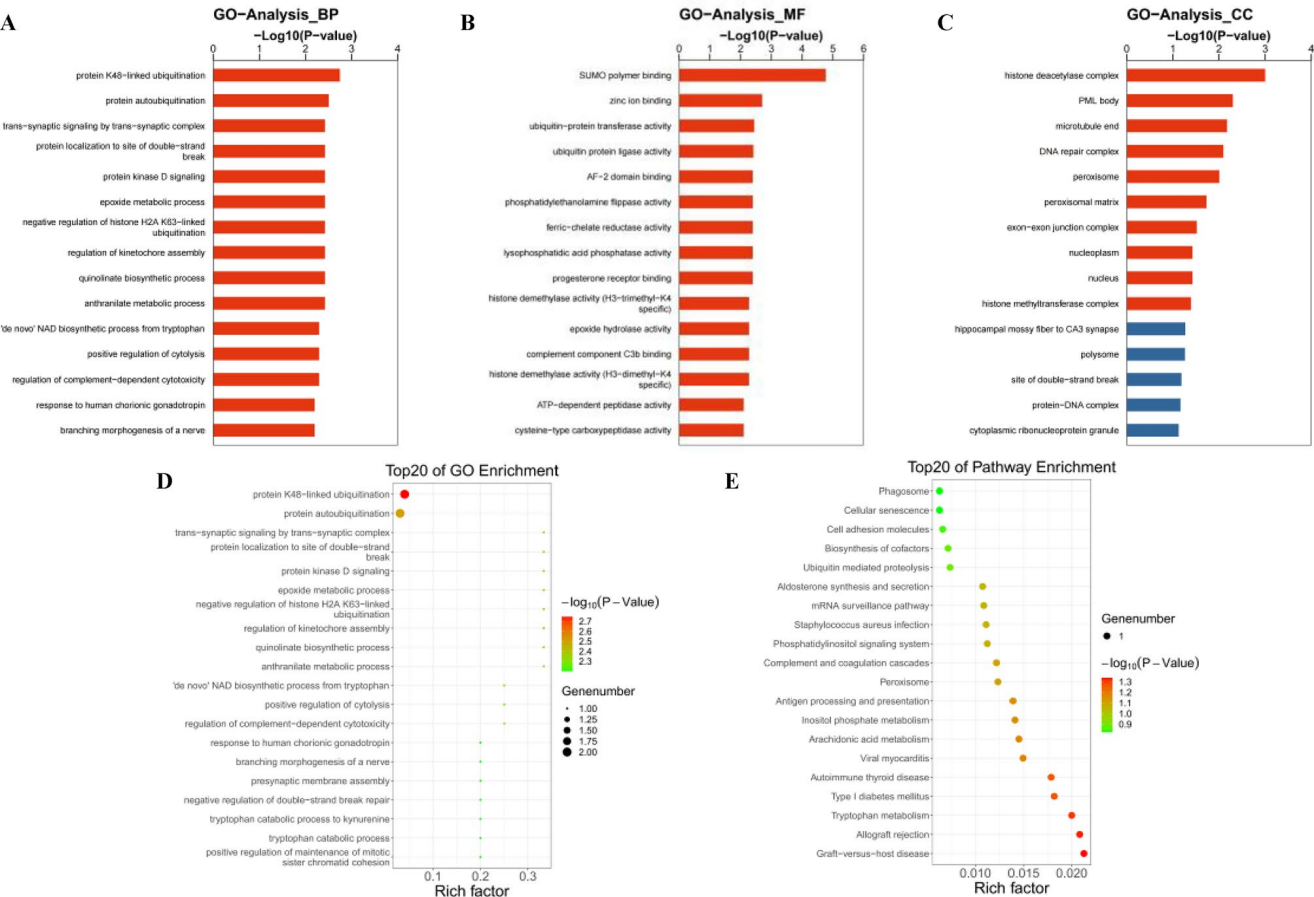
GO and KEGG analyses of differential circRNA host genes in the liver of PM_2.5_-exposed rats. (**A**) Biological process analyzed by GO enrichment. (**B**) Molecular function of GO enrichment analysis. (**C**) GO enrichment analysis of cellular components. The horizontal axis represents the *P*-value, indicating the significance of the enrichment of the input target gene for each GO term. The vertical axis represents the GO term. (**D**) Top 20 GO terms. The dot color represents different *P*-values and the dot size represents the number of genes. (**E**) Top 20 KEGG-enriched pathways for differentially expressed circRNAs. The dot color represents different *P*-values and the dot size represents the number of genes.

We performed GO functional analysis of DEcircRNA host genes and analyzed three GO categories, showing the top 15 biological processes (BP), molecular functions (MF), and cellular components (CC; Fig. 6A–C). Three GO terms were observed. The results indicate activities of PM_2.5_-induced DEcircRNAs such as protein ubiquitination, binding to zinc ions, and participating in cycloperoxide metabolism. The top 20 significant enrichment terms of the GO analysis are presented in Fig. 6D. The results show that protein ubiquitination may be associated with liver injury due to PM_2.5_ exposure. The top 20 pathways based on KEGG pathway analysis, including amino acid metabolism, peroxisomes, and immune pathways, are shown in Fig. 6E, indicating that these pathways may be involved in liver injury due to PM_2.5_ exposure.

### Analysis of circRNA-miRNA interaction

**Figure 7:**
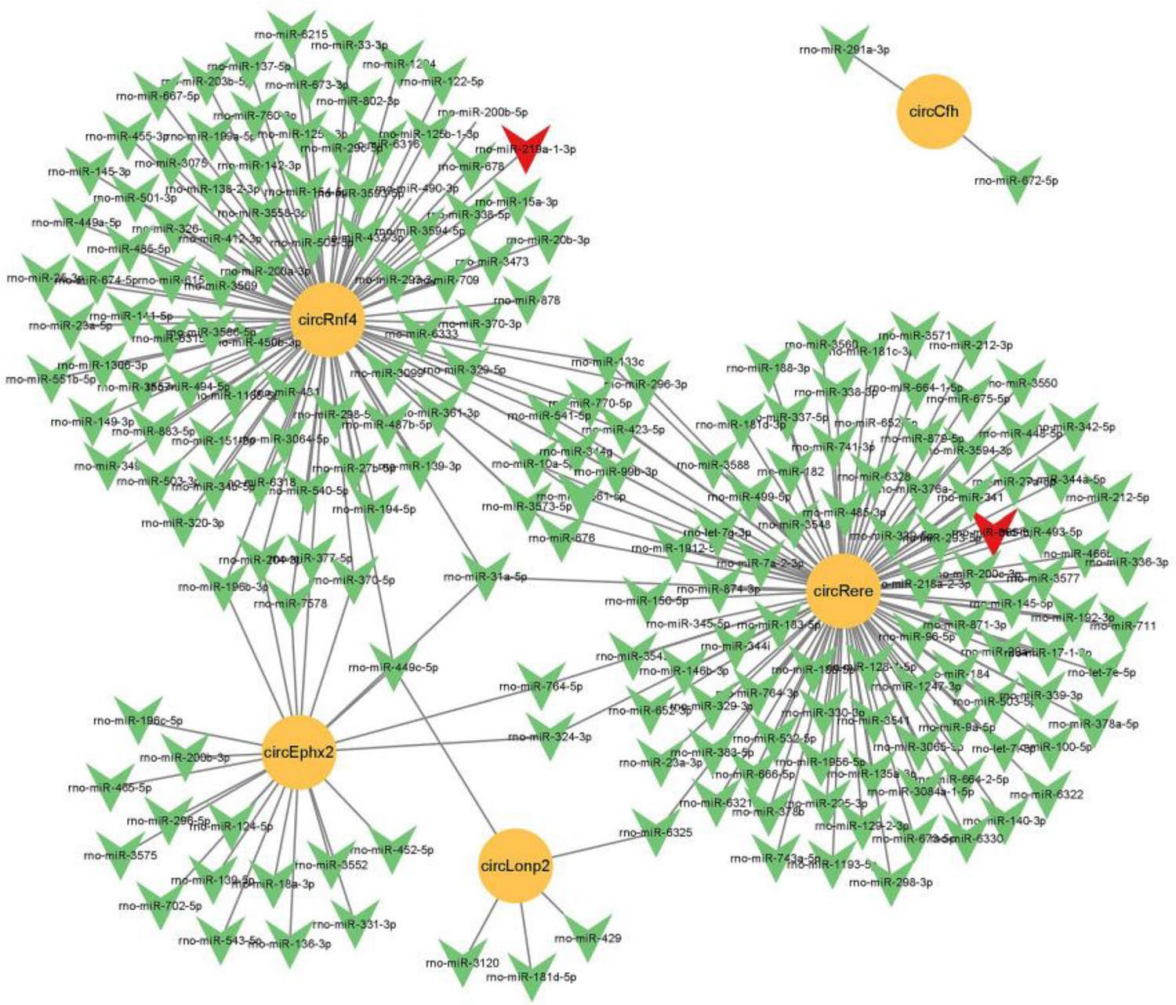
circRNA-miRNA interaction network. Green V nodes represent miRNAs, yellow circular nodes represent circRNAs, and the red V node indicates that the predicted miRNA has the same miRNA as the differential miRNA obtained by sequencing.

CircRNAs are a class of ncRNAs that function as miRNA sponges. In order to further verify the mechanism of DEcircRNA in liver injury induced by PM_2.5_ exposure, according to GO and KEGG results, six DEcircRNAs related to protein ubiquitination, Zn^2+^ binding, and peroxisome pathways were selected to predict miRNA binding sites and construct the circRNA-miRNA interaction network (Fig. 7). The results showed that circCfh had 2 miRNA nodes, circEphx2 had 23 miRNA nodes, circRere had 105 miRNA nodes, circLonp2 had 5 miRNA nodes, and circRnf4 had 96 miRNA nodes. Among all the predicted miRNAs, two miRNAs (rno-miR-219a-1-2p and rno-miR-365-5p) were found to be identical to the differential miRNAs obtained by sequencing (Supplementary Table S3). The results showed that circRNA could participate in the liver injury induced by PM_2.5_ exposure by binding multiple miRNAs.

### Identification of DEcircRNAs by RT-qPCR

**Figure 8:**
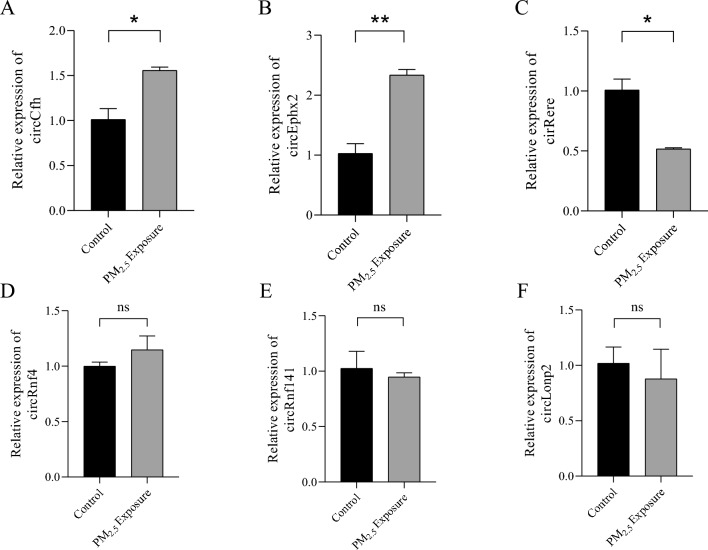
RT-qPCR results of differentially expressed circRNA. (**A-F**) RT-qPCR results of circCfh, circEphx2, circRere, circRnf4, circRnf141 and circLonp2. *: *P* < 0.05; **: *P* < 0.01; ns: *P* > 0.05.

The above 6 DEcircRNAs were selected for RT-qPCR to verify their expression in liver tissues. Results As shown in Fig. 8, compared with the control group, the expression of circCfh and circEphx2 in the liver tissue was up-regulated and the expression of circRere in the liver tissue was down-regulated in the PM_2.5_ exposure group, which was consistent with the sequencing results. There was no difference in the expression levels of the other three circRNAs between the two groups.

## Discussion

In this study, we established an experimental model of the PM_2.5_ exposure of the respiratory tract of rats. Liver tissue samples were collected after 16 weeks of exposure. The results of ICP-MS showed that Mn, Cu and Ni were enriched in liver. After HE staining of liver tissue sections, evident abnormalities in the hepatic tissue structure were observed in the PM_2.5_-exposure samples. Transmission electron microscopy analysis showed that the morphology and quantity of hepatocyte mitochondria in PM_2.5_-exposure group were significantly changed, accompanied by changes in mitochondrial function, including increased intracellular ROS content, decreased mitochondrial transmembrane potential, and up-regulated expression of antioxidant enzymes (SOD1, HO-1). After high-throughput sequencing, 10 circRNAs were found to be significantly downregulated, while 17 were upregulated. Functional enrichment and pathway analysis indicated that the host genes of aberrant circRNA in PM_2.5_-exposed livers were primarily associated with processes related to protein ubiquitination, zinc ion binding, peroxisome function, and mitochondrial regulation. These findings suggest that the mechanism underlying liver injury induced by PM_2.5_-exposure may be associated with mitochondrial impairment resulting from the presence of heavy metal constituents.

The mechanism by which PM_2.5_ exposure causes mitochondrial damage is not fully understood, but some studies have shown that it may be related to the heavy metal components in PM_2.5_. Heavy metals are an important component of PM_2.5_ and the main substance that damages the body. Studies have shown that after respiratory tract exposure to PM_2.5_, its heavy metal components can be widely enriched in various tissues and organs, such as the lungs, heart, brain, liver and kidneys. Further, these heavy metal components can damage the liver and other organs by inducing oxidative stress^13^. In this study, the content of Mn, Cu and Ni in the PM_2.5_ exposure group was higher than that in the control group, and there were differences. Studies have confirmed that Mn, Cu and Ni can stimulate liver cells or liver cancer cells to produce excessive ROS, induce oxidative stress, destroy the morphology and function of mitochondria, and eventually lead to liver damage^36,37^. Liu et al. ^38^ found that Cu-induced hepatocyte apoptosis was the result of mitochondrial transmembrane permeability damage caused by Cu-induced ROS overproduction and weakened antioxidant function. In addition, Ni also has hepatotoxicity, which can lead to apoptosis of hepatocytes by inducing ROS production and destroying mitochondrial transmembrane potential, and with the addition of Mn, this effect will play a greater role^39,40^.

This study observed that prolonged PM_2.5_ exposure caused alterations in the structure of the liver tissue of rats, such as the destruction of the liver lobular structure, the disarrangement of liver cells, and inflammatory cell infiltration, suggesting that PM_2.5_ exposure via the respiratory tract can indeed cause liver damage. Transmission electron microscopy showed that the internal structure of the liver cells of the PM_2.5_-exposed group was disturbed, which was mainly manifested as mitochondrial structure disorder, including a blurring of the internal mitochondrial structure, the fusion for the inner and outer membranes, the disappearance of the membrane gap, the dissolution of the mitochondrial ridge, and the merging of two mitochondria into one. It is suggested that the mechanism by which PM_2.5_ exposure causes liver damage may be closely related to mitochondrial damage. This is consistent with the results of Jiang et al^41^, who showed that PM_2.5_ exposure can lead to tissue and organ damage in heart tissues, and further studies that suggest that it is closely related to mitochondrial damage, which is consistent with the results of this study. The production of ROS is an important factor leading to oxidative stress and mitochondrial dysfunction^42^. We found that the reactive oxygen species level of rat liver cells increased and the mitochondrial transmembrane potential decreased after exposure to PM_2.5_, which was consistent with the study of Qiu and Zhu et al.^20,43^, and the expression of antioxidant enzymes SOD1 and HO-1 genes was up-regulated. This suggests that the liver may respond to oxidative stress caused by PM_2.5_ exposure by increasing the expression of antioxidant oxidase genes.

To further investigate the mechanism by which PM_2.5_ exposure causes mitochondrial damage in hepatocytes, this study conducted an in-depth assessment through circRNA high-throughput sequencing combined with a bioinformation analysis (including GO and KEGG analyses). A total of 5,585 circRNAs were identified. Further analysis screened out 27 DEcircRNAs, including 10 significantly down and 17 significantly up-regulated circRNAs. These results showed that PM_2.5_ exposure could lead to changes in the expression of multiple circRNAs. Functional annotation and pathway analysis of DEcircRNA showed that it was mainly enriched in the protein-ubiquitin system, zinc ion binding, peroxisome, and other pathways. In contrast to this study’s results, Chen et al.^44^ pointed out that circRNA in the hippocampus of rat brain tissue was mainly enriched in vesicle-mediated transport from the endoplasmic reticulum to the Golgi apparatus, histidine biosynthesis process, and MAPK signaling pathways among others. The reason for these inconsistent results may be the specificity of the tissues and organs.

We have demonstrated that DEcircRNAs primarily participate in the regulation of three key processes (protein ubiquitination, Zn^2+^ binding, and peroxisome pathways). Importantly, these processes have been extensively linked to the occurrence of oxidative stress^45–47^, which primarily occurs within mitochondria and, is primarily caused by the production of ROS. Mitochondria also serve as the principal sites of ROS generation, and an excessive production of ROS can result in mitochondrial damage. Furthermore, impaired mitochondria can elicit a feedback loop by generating more ROS, thereby exacerbating mitochondria impairment. The liver has been identified as the primary organ involved in the Zn^2+^ metabolism, playing a crucial role in zinc homeostasis. Zinc, predominantly present as zinc oxide nanoparticles within PM_2.5_, is absorbed by the human body in the form of Zn^2+^. However, exposure to zinc oxide nanoparticles can lead to an excessive generation of free radicals and subsequent oxidative stress. Irrespective of the mode of exposure, excessive intake of zinc has been shown to induce liver injury^48–51^. Furthermore, Zn^2+^ has been established as a key regulator in preventing mitochondrial injury through extracellular kinase signaling pathways and limiting superoxide production^52^. Nevertheless, some studies have suggested that mitochondria may be susceptible to cytotoxic effects induced by Zn^2+^, with varying concentrations causing distinct forms of mitochondrial damage. Lower concentrations result in mitochondria swelling, while higher concentrations disrupt the permeability of the inner membrane towards H^+^ and K^+^ ions^53^. Secondly, the potential role of the peroxisome^54^ pathway in PM_2.5_- exposure-induced liver injury has also been reported. It is worth noting that peroxisome plays an important role in the generation and metabolism of free radicals^55^. Wang et al.^56^ predicted potential targets of arsenic-induced hepatotoxicity, finding that arsenic interferes with the metabolism of glutathione in the liver, and that Epoxide hydrolase 2 (Ephx2) is a key target involved in arsenic-induced hepatotoxicity. Interestingly, our results showed that Ephx2 is a chr15:42766794–42766683 host gene, which may further suggest the important role of circRNA in PM_2.5_-exposure-induced liver injury. Peroxisomes are critical to mitochondrial health because some genetic disorders, such as Zellweger Spectrum Disorders, result from peroxisomal defects and dysfunction associated with hepatic mitochondrial dysfunction^57^. Chi et al. confirmed the important role of the peroxisome in mitochondrial function and structure by knocking out the peroxisome gene Pex16, which may be related to the tricarboxylic acid cycle (TCA) and electron transport chain^58^. Studies have shown that PM_2.5_ exposure can inhibit mitochondrial dysfunction in HepG2 cells, which is characterized by a reduced energy supply of the TCA cycle and oxidative phosphorylation^59^. Finally, the relationship between protein ubiquitination and mitochondria has been reported. E4 ubiquitin ligase promotes the extension of ubiquitin chains in nematodes and the degradation of proteasomes, resulting in mitochondrial damage, which in turn induces the production of ROS, promoting the phosphorylation of certain nucleoprotein oncogenes for ubiquitination^60,61^. Our study found that Rnf4 and Ubr5 were involved in the protein ubiquitination and Zn^2+^ pathways, while Ephx2 was involved in the peroxidase pathway, but their biological significance has not been assessed in detail and further research is needed. CircRNA sequencing and bioinformation analysis further confirmed that the liver damage caused by PM_2.5_-exposure is due to its heavy metal components causing oxidative stress that damages the structure and function of mitochondria.

Furthermore, to gain deeper insights into the underlying mechanism of liver injury induced by PM_2.5_ exposure, we conducted predictive analyses on circRNA-miRNA interactions. The results of a previous study showed that circRNAs act as miRNA sponges and participate in gene regulation^62^. For example, has-circ-0126672 can sponge miR-145-5p, miR-186-5p, miR-548c-3p, miR-7-5p, miR495-3p, miR-203a-3p, and miR-21, affecting various factors associated with coronary heart disease^63^. Similarly, some miRNAs potentially play roles in PM_2.5_-induced liver injury. MicroRNA-26a-CD36 is considered a critical regulator of PM_2.5_-induced abnormal lipid metabolism in hepatocytes^64^. In order to explore the interaction between circRNA and miRNA, we constructed a circRNA-miRNA interaction network. The results showed that one circRNA could bind to multiple miRNAs, and similarly, one miRNA could bind to multiple circRNAs. In addition, two miRNAs (rno-miR-219a-1-3p and rno-miR-365-5p) predicted by the interaction network were consistent with the differentially expressed miRNAs obtained by sequencing. miR-219a-1-3p has been shown to affect the osteogenic ability of dental pulp stem cells by targeting heat shock protein B8^65^ and its role in iron death^66^. miR-365-5p has been confirmed to be associated with hypertrophy of white adipose tissue^67^, maintenance of bone tissue homeostasis^68^, and neuronal apoptosis^69^. However, these two miRNAs have not been reported in studies on liver-related diseases after PM_2.5_ exposure, and they still need to be explored.

Lastly, the limitations of this study should be noted. First, we used animal experiments to simulate PM_2.5_ exposure to evaluate the liver damage it causes, which may deviate from its effects on humans. Second, the tracheal instillation method used in this study for PM_2.5_ respiratory exposure is a commonly used method in animal experiments, but it again introduces a certain deviation from the actual mechanism of continuous PM_2.5_ exposure in humans. Finally, this study used high-throughput RNA sequencing to identify DEcircRNAs in liver tissue after PM_2.5_ exposure and combined this with bioinformatics analyses to predict their biological functions. However, further research is needed to determine the actual function of DEcircRNAs in organisms.

## Conclusions

In summary, our study found that long-term exposure to PM_2.5_ through the respiratory tract can cause liver injury in laboratory animals, mainly manifested as a dysfunction of the mitochondrial structure in liver cells. Its mechanism of action may be related to the heavy metal components of PM_2.5_, which affect protein ubiquitination and mitochondrial peroxidase function, resulting in oxidative stress and thus mitochondrial dysfunction and damage. Therefore, this study provides a new theoretical basis for exploring the molecular mechanism of liver injury caused by PM_2.5_ exposure.

### Supplementary Information

Supplementary Information.

## Data Availability

The datasets generated during and/or analyzed during the current study are available from the corresponding author on reasonable request.
