# Supplementary material for: Long-term exposure to PM2.5 leads to mitochondrial damage and differential expression of associated circRNA in rat hepatocytes
Source: Sci Rep. 2024 May 24;14:11870. doi: 10.1038/s41598-024-62748-y (PMC11126672; doi:10.1038/s41598-024-62748-y)
Supplement: Supplementary file 1 — Supplementary Information. [file 41598_2024_62748_MOESM1_ESM.docx]

**Long-term exposure to PM_2.5_ leads to mitochondrial damage and differential expression of associated circRNA in rat hepatocytes**

***Ying Liu^a^, Jing Li^a^, Yican Xiong^b^, Chaochao Tan^a^, Cunyan Li^a^, Youde Cao^a^, Wanying Xie^a^, Zhonghua Deng^a,*^***

*^a^* Department of Medical Laboratory, Hunan Provincial People's Hospital, the First Affiliated Hospital of Hunan Normal University, Changsha, 410005, PR. China

*^b^* Department of Ophthalmology & Stomatology, Hunan Provincial People's Hospital, the First Affiliated Hospital of Hunan Normal University, Changsha, 410005, PR. China

*^*^* Corresponding Author: Department of Medical Laboratory, Hunan Provincial People's Hospital, the First Affiliated Hospital of Hunan Normal University, Changsha, 410005, PR. China. Email:[speed1027@163.com](mailto:speed1027@163.com)


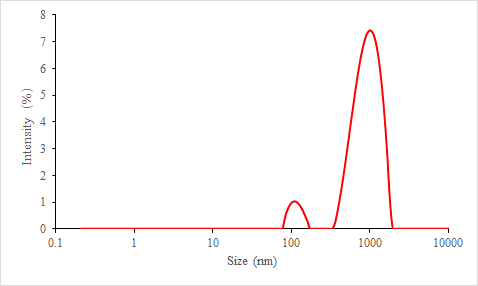


**Fig. S1 Particle size analysis of PM_2.5_**

**Fig. S2 HE and transmission electron microscopy results of rat liver. A** HE staining results of rat liver. Magnification of 100x, 200x and 400x. **B** Mitochondria of rat liver. Magnification of 1500x, 7000x and 15000x.


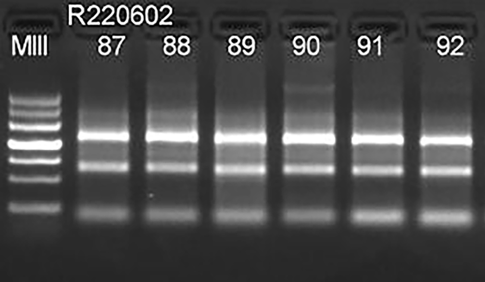


**Fig.S3 Detection of RNA integrity by agarose gel electrophoresis.** The three bright lines from the top down represent 28S rRNA, 18S rRNA, and 5S rRNA, and the brightness of 28S rRNA is about twice that of 18S rRNA, indicating that the RNA has good integrity.

**Fig.S4 Agilent 2100/2200 Bioanalyzer to detect RNA integrity of six tissue samples. A-F** The RNA inspection results of D1, D2, D3, S1, S2, and S3 samples respectively. The three peaks in the figure represent 5S rRNA, 18S rRNA, and 28S rRNA from left to right.

**Table S1 Primer sequences for RT-qPCR**

| **Gene** | **Forward Primer (5’ to 3’)** | **Reverse Primer (5’ to 3’)** |
| --- | --- | --- |
| SOD1 | TGGGGACAATACACAAGGCTG | ATGCCTCTCTTCATCCGCTG^[1]^ |
| HO-1 | ACAGCACTACGTAAAGCGTCTCCA | CATGGCCTTCTGCGCAATCTTCTT^[2]^ |
| circRnf141 | TTCTTGGGAGAGTTGCTGAAC | TACAGACCACCCGTACGACC |
| circCfh | CGGGTTTACAACGCCTTCAC | GAGGCATGGAACTTCAGGCT |
| circRere | ATCAAGAACCGGGAGCTCTTC | GTCCACTCGGGCTTTAAATTCAC |
| circRnf4 | CCAGAAGCGAACTCGGGAAA | CCACGATTTCATCTCCAACGG |
| circLonp2 | GCTGACTCCTCAGCAGATCC | TGCAACTTTCACTGCAACGG |
| circEphx2 | TGGCCAGTTTGAACACTCCAT | GCTGATAGTTGAAAACTGGGATCG |
| *β*-actin | TGTCACCAACTGGGACGATA | GGGGTGTTGAAGGTCTCAAA |

**Table S2 Nine metals in water, food and PM_2.5_**

| **Metal** | **Water(μg/kg)** | **Food(mg/kg)** | **PM_2.5_(mg/kg)** |
| --- | --- | --- | --- |
| Mn | 2.04±0.034 | 187.0±1.379 | 640.0±1.992 |
| Cu | 1.54±0.043 | 19.84±0.255 | 505.0±0.271 |
| As | 3.94±0.014 | 0.47±0.021 | 83.19±0.364 |
| Ni | 0.47±0.014 | 2.73±0.059 | 59.16±0.396 |
| V | 0.60±0.003 | 0.34±0.001 | 87.78±0.552 |
| Cd | 0.103±0.001 | 0.076±0.003 | 54.70±0.264 |
| Pb | 0 | 0.17±0.001 | 576.0±0.600 |
| Co | 0 | 2.97±0.035 | 12.83±0.795 |
| Hg | 0 | 0 | 0.65±0.037 |

**Table S3 Differentially expressed miRNAs**

| **ID** | **log_2_FC** | ***P*-value** |
| --- | --- | --- |
| rno-miR-632 | -3.268146773 | 0.03535542 |
| rno-miR-135b-5p | -1.340980381 | 0.035071655 |
| rno-miR-365-5p | -2.834200765 | 0.000666443 |
| rno-miR-219a-1-3p | -1.994975302 | 0.007577701 |
| rno-miR-463-3p | -2.151894857 | 0.027235228 |
| rno-miR-347 | -1.646180161 | 0.011161922 |
| rno-miR-875 | -1.58057657 | 0.027260842 |
| rno-miR-297 | -1.703940808 | 0.017813061 |

[1] S Y, W S, Q L, et al. Nrf2 enhances the therapeutic efficiency of adipose-derived stem cells in the treatment of neurogenic erectile dysfunction in a rat model [J]. *Basic Clin Androl*, 2023, 33(1): 023-00214.

[2] LC S, ID O, JM D A C, et al. Kisspeptin-10 Improves Testicular Redox Status but Does Not Alter the Unfolded Protein Response (UPR) That Is Downregulated by Hypothyroidism in a Rat Model [J]. *Int J Mol Sci*, 2024, 25(3).
